# Children’s Attention to Mother and Adolescent Stress Moderate the Attachment-Depressive Symptoms Link

**DOI:** 10.5334/pb.550

**Published:** 2020-09-04

**Authors:** Guy Bosmans, Magali Van de Walle, Patricia Bijttebier, Simon De Winter, Joke Heylen, Eva Ceulemans, Rudi De Raedt

**Affiliations:** 1 Clinical Psychology, KU Leuven, Leuven, BE; 2 School Psychology and Development in Context, KU Leuven, Leuven, BE; 3 Parenting and Special Education, KU Leuven, Leuven, BE; 4 Quantitative Psychology and Individual Differences, Leuven, BE; 5 Experimental Clinical and Health Psychology, Ghent University, Gent, BE

**Keywords:** Attachment, Middle Childhood, Adolescence, Depressive Symptoms, Information Processing

## Abstract

The breadth of children’s attentional field around their mother determines whether securely or insecurely attached children are at risk to develop depressive symptoms when confronted with distress in adolescence. To test this effect longitudinally, we measured children’s (*M*_*age*_ = 10.93; *N* = 109) baseline attentional breadth around their mother, attachment status (combining attachment coherence, secure base script knowledge, and self-reported trust), and self-reported depressive symptoms. One and two years later, we measured self-reported distress and depressive symptoms. We tested three-way interactions between attentional breadth × attachment × distress on changes in depressive symptoms. This three-way interaction was marginally significantly linked with changes in depressive symptoms from baseline to year 1, and significantly with changes in depressive symptoms from baseline to year 2. Results pointed to the protective role of a narrow attentional field around the mother in middle childhood for securely attached children who are confronted with distress later in life.

The development of depressive symptoms is rooted in childhood, but once it emerges in adolescence and translates to depressive disorders, the psychosocial and economic impact is substantial both for affected individuals as for the society (World Health Organization, 2001). Hence, longitudinal research on childhood precursors of the development of depressive symptoms is critical to understand which interventions could help promote mental health. One childhood factor often proposed to be related to the development of depressive symptoms is children’s (lack of) trust in caregiver support, or (in)secure attachment (Bowlby, 1969; Dujardin et al., 2016). Although longitudinal research on attachment and depressive symptoms is scarce, previous cross-sectional and longitudinal research points at significant, yet modest direct associations (Madigan et al., 2016). This raises the question whether moderators might affect the direct association between attachment and depressive symptoms and whether one can identify which (in)securely attached children are more or less vulnerable to develop depressive symptoms. Prior research has pointed to distress (Dujardin et al., 2016) and to attachment-related information processing biases moderating the link between attachment and symptoms of psychopathology (Bosmans et al., 2013; Claes et al., 2016; De Winter et al., 2018; Van de Walle et al., 2017). Research suggests that both moderators might interact as well and that less securely attached children’s risk to develop depressive symptoms in adolescence might depend on whether they are exposed to distress and on whether or not they more easily focus their attention on mother during distress. Therefore, in the current study, we tested this hypothesized three-way interaction effect. Specifically, we tested whether the breadth of children’s attentional field around mother determines whether (in)securely attached children are more or less at risk to develop depressive symptoms over time depending on exposure to distress in adolescence.

## Attachment and the Development of Depressive Symptoms

According to attachment theory, children become securely attached if they learn that they can trust in primary caregivers’ (mostly parents) support during distress. More securely attached children more easily seek caregiver support during distress, which promotes adaptive development (Bowbly, 1969). Instead, if children experience that support is inconsistent or consistently absent, trust decreases. Consequently, they become insecurely attached, which impairs support seeking during distress (Bowlby, 1969; Bosmans et al., 2020). Children who are less securely attached can develop two different insecure attachment styles. Some display preoccupied attachment behavior which means that they seek support but constantly fear to be disappointed by their parent, which elicits frustration and anger. Others display dismissing attachment behavior, which means that they refuse to seek support, dismiss any need for support and instead avoid emotional stimuli. Although attachment development is prominent in the early years, accumulating research shows that attachment is subject to change contingent upon changes in the relational environment throughout the life-span (Bosmans et al., 2020), with substantial shifts having been observed throughout middle childhood and during the transition to adolescence (Waters et al., 2019). This renders the study of attachment at later ages highly important to understand the development of psychopathology such as depressive symptoms.

Attachment is a complex construct consisting of different components (Bosmans & Kerns, 2015; Bosmans et al., 2020): (a) children’s level of self-reported trust in caregiver’s support (Ridenour et al., 2006), (b) the extent to which children have a cognitive script about a caregiver who is available and responsive when the child encounters a distressing event and who helps the child get back on track (secure base scriptedness) (H.S. Waters & Waters, 2006), and (c) the extent to which children have a coherent discourse about experiences and relationships with caregivers (Target et al., 2003). Children performing positively on all three indicators are more likely securely attached, whereas worse performance indicates more insecure attachment. There is increasing awareness that each indicator reflects a component of the complex attachment construct and that all components should be considered to fully grasp attachment and its links to outcomes like depressive symptoms (Bosmans & Kerns, 2015; Steele, 2015).

The link between middle childhood attachment and depressive symptoms has mainly been investigated in cross-sectional studies, but has been confirmed longitudinally as well (Brumariu & Kerns, 2010). However, the effect size of this link appears to be modest. Different meta-analyses found effect sizes ranging from *d* = .15 (Groh et al., 2012) to *d* = .58 (Madigan et al., 2016) suggesting that not all insecurely attached children develop depressive symptoms. The modest size of this effect might be due to the fact that insecure attachment is not a synonym of psychopathology (Bosmans et al., 2020). Instead, these children are more at risk to develop depressive symptoms because they can benefit less from the buffering effect of support seeking when they experience distress (Cassidy, 1994). In other words, this suggests that the link between attachment and depressive symptoms needs to be studied accounting for the moderating effect of distress.

## The Moderating role of Distress

The experience of distress plays a critical role in the development of depressive symptoms (e.g., Tennant, 2003). Distress is an emotional reaction experienced as suffering in the presence of objective or subjective stressors. Accumulated over time, stress can lead to mental health problems of which depressive symptoms are one widely acknowledged outcome (Drapeau et al. 2012). Distress leads to enhanced secretion of stress-related hormones such as cortisol, and if this occurs chronically, it further impairs adequate endocrinological functioning, resulting in depressive symptoms in some but not all children (Hammen, 2018). This research suggested that also the strength of the association between distress and change in depressive symptoms is moderated by other factors. One important moderator of the association between stress and the development of depression during the transition from middle childhood to adolescence is social support (Wang et al., 2014). This is more often solicited for by more securely attached children (Bosmans et al., 2015).

In a longitudinal study, Dujardin et al. (2016) found support for the hypothesis that the interaction between attachment and distress explains the development of depressive symptoms over time. They observed that children who were less securely attached waited longer to seek maternal support during a distressing task. However, those children only developed depressive symptoms in adolescence if they were exposed to distress. This points at distress as a moderator of the association between attachment and the development of depressive symptoms over time.

## The moderating role of children’s attentional breadth around the mother

Accumulating research suggests that attachment-related information processing biases further moderate the association between attachment and depressive symptoms (e.g., Bosmans et al., 2013). Biases in children’s processing of information regarding their mother as a resource during distress have been associated to differences in children’s (in)secure attachment (e.g., Bosmans et al., 2019). It has been proposed that these biases develop through classical and operant learning processes (Bosmans et al., 2020) and further shape which expectations children derive from support-related learning experiences (Verhees et al., 2019). Attachment-related information processing biases have been demonstrated at the level of attachment-related memory biases (Dujardin et al., 2014), interpretation biases (De Winter et al., 2018), and attentional biases (e.g., Bosmans et al., 2009).

The most studied attentional bias so far is the breadth of children’s attentional field around their mother (Bosmans et al., 2009). This bias refers to the extent to which children are able to attentionally process information that is presented around mother. Children who are less able to detect stimuli presented further away from the mother have a narrower attentional field around her. To measure children’s attentional field around their mother, a picture of the mother together with a target stimulus are presented at a presentation time that prevents eye saccades (32 ms). This way, the breadth of the attentional field around the mother refers to the pre-attentive stages of information processing, more automatically triggered by the motivational or emotional relevance of the mother as a stimulus (Bosmans et al., 2017; Derryberry & Tucker, 1994).

**Figure 1:**
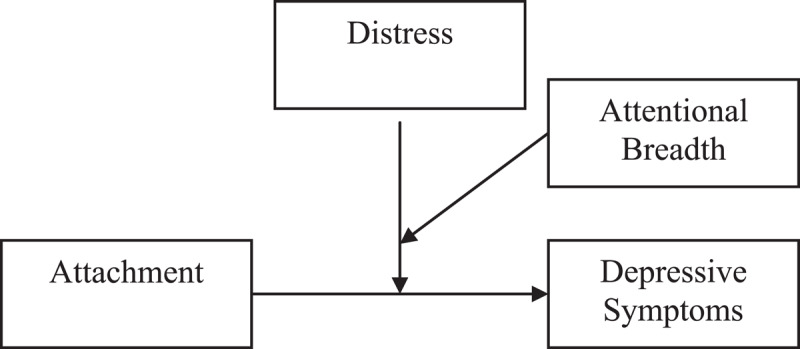
Theoretical Model.

Early attachment-related attentional breadth research suggested that differences in attentional breadth reflect differences in children’s attachment with more insecurely attached children having a narrower attentional field around mother than more securely attached children (Bosmans, 2009; 2015). However, an accumulating number of studies showed that a narrower attentional field around mother serves as a protective factor for more securely attached children, but as a risk factor for more insecurely attached children. This effect was found for emotional and behavioral problems (Bosmans et al., 2013; Van de Walle et al., 2016) and for non-suicidal self-injury (Claes et al., 2016). It has been proposed that having a narrower attentional field around the mother may be helpful during distress for more securely attached children because it can facilitate support seeking behavior. Instead, for more insecurely attached children, a narrower attentional field around mother might reflect a stronger inclination to ruminate about the option to seek proximity and a more elaborate evaluation of the availability of the mother for support (e.g., Van de Walle et al., 2017). This could also delay proximity seeking during distress (Bosmans et al., 2015). Although this interpretation suggests that there should be a three-way interaction between attachment, distress, and attentional breadth that explains the development of depressive symptoms (see Figure 1), this hypothesis was never directly tested.

## The Current study: Testing the Attachment × Distress × Attentional Breadth Interaction

The current study aimed to test this hypothesis using a longitudinal research design comprising three measurement waves, with a one-year time interval between each wave. We started the study towards the end of middle childhood and followed up the development of depressive symptoms through the transition to adolescence. During this transition, the prevalence of depressive symptoms dramatically increases (Tram & Cole, 2006), which makes this a valuable period in life to study whether the proposed three-way interaction can be linked to the longitudinal development of depressive symptoms. Finally, we focused on children’s attachment towards mother, because at this age, she remains the primary attachment figure (Vandevivere et al., 2015).

Attachment was measured at baseline, combining children’s performance on the three relevant components of attachment (self-reported trust, secure base scriptedness, and narrative coherence) that are to date mostly used in middle childhood attachment research. These three measures were used to calculate a continuous common factor score. This approach creates a multi-method score of (more or less) secure attachment that reflects the extent to which children report to trust in maternal support, have a Secure Base Script (SBS), and are able to coherently discuss attachment experiences. This way, we ensured to have an attachment measure that largely reflects the broader attachment construct (Bosmans & Kerns, 2015). Children’s attentional breadth around the mother was measured with a computerized task (Bosmans et al., 2009; 2013; 2017; Claes et al., 2016; Van de Walle et al., 2017). Distress was measured at both follow up waves, focusing on daily distressing events that occurred within three months before the assessment. Daily hassles seem most strongly associated with depressive symptoms, and they are more prevalent in community samples compared to life events (Hammen, 2005; Low et al., 2012; Monroe & Reid, 2008). Finally, depressive symptoms were measured at baseline and at both follow up waves using self-report, because children have shown to be the most valid reporter of internalizing problems (Angold et al., 1987).

In the current study, the three-way interaction between middle childhood attachment, attentional breadth around the mother, and distress during follow up was tested on changes in depressive symptoms by using depressive symptoms at follow up as dependent variable and using baseline depressive symptoms as control variable. Further, attachment was included as independent variable, and distress and attentional breadth as moderators (see Figure 1). This interaction was tested twice, separately predicting change in depressive symptoms from baseline to Time 2 and predicting change in depressive symptoms from baseline to Time 3, because we expected this effect to become stronger as children progressively transition in adolescence and become more vulnerable to develop depressive symptoms.

## Method

### Participants

In this study, 157 children (52% girls) from 16 Belgian primary schools aged 9 to 12 years old (*M* = 10.93, *SD* = 0.88) participated at Time 1 (T1). This is the same sample as in Study 2 of Van de Walle et al. (2016) and as in Van de Walle et al. (2017). Most of the children lived with both parents (76%), with mother as a primary caregiver during the first three years of life (97%). With regard to education, 21% of the mothers had an elementary school or high school degree, 36% did specialization studies after high school, and 43% had a university degree (for fathers this was 31%, 24%, and 46% respectively). One year follow-up data at Time 2 (T2) and two year follow-up at Time 3 (T3) were available for respectively 146 and 133 children (93% and 85% of the original sample). The children who dropped out did not differ significantly from the original sample on the T1 variables (age, sex, attentional narrowing around mother, attachment, and depressive symptoms) in a multivariate analysis of variance, *F*(7,128) = 0.48, *p* = 0.845, and in independent samples t-tests.

### Measures

#### Attentional narrowing around mother

**Figure 2:**
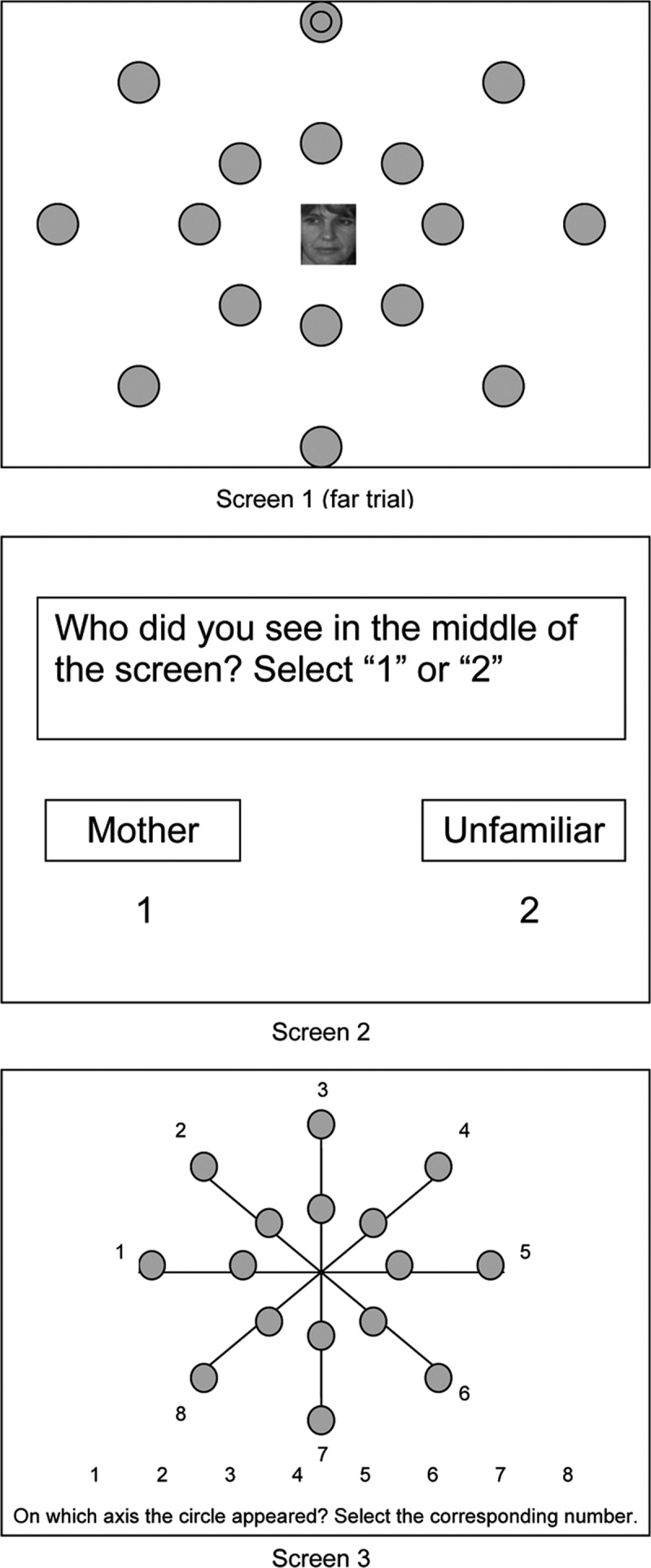
Stimulus presentation of the ABT. From “Attachment security and attentional breadth toward the caregiver in middle childhood” by G. Bosmans, C. Braet, E. Koster and R. De Raedt, 2009, *Journal of Clinical Child & Adolescent Psychology, 38*, p. 875.

The Attentional Breadth Task (ABT; full description available in Bosmans et al., 2009) is a computerized experimental task to measure attentional narrowing around mother. This task involves a dual-task paradigm. Children are seated in front of a 19” CRT-computer screen at a distance of 27 cm using a chin rest. In each trial, a picture of the mother’s or an unfamiliar woman’s face is presented during 34 ms in the center of the computer screen. There are 10 pictures of 10 different unfamiliar women and 10 different pictures of the mother. Simultaneously with the picture, a smaller black circle appears close to or far from this picture. This target is presented in one of 16 grey dots, which are arranged in pairs of two on eight imperceptible axes. At each trial, children have to identify the picture first and thereafter they have to indicate on which of eight axes the target was presented. An example of a trial is shown in Figure 2. By combining two picture types and two distances, there are four target presentation categories: mother close, mother far, unfamiliar woman close, and unfamiliar woman far. Each category contains 16 test trials that are randomly presented per block. There are 2 blocks, separated by a short break. In total, 128 test trials are presented. Beforehand, children complete eight practice trials at 250 ms, and 16 practice trials at 34 ms.

To calculate the extent of attentional narrowing around mother, several steps are taken. First, all trials in which the child identified the picture incorrectly are deleted. This way, correct identification of the target cannot be due to coincidental or purposeful gazing at the target’s location, as the child is looking at the center of the computer screen. Then, the proportion of correct responses is calculated for the four target presentation categories. For both mother and unfamiliar women trials, the proportion of correct responses on far trials is subtracted from the proportion of correct responses on close trials. This results in two attentional narrowing indices (ANI). The attentional narrowing effect (ANE) is calculated by subtracting the ANI for the unfamiliar women from the ANI for the mother. This way, higher levels of ANE imply a narrower attentional field around mother compared to unfamiliar women. In prior research, ANE predicted children’s support seeking behavior during distress (Bosmans et al., 2015).

### Attachment

#### Trust Scale

Children filled out the mother-version of the Trust Scale of the self-report questionnaire People In My Life (PIML; Ridenour et al., 2006) at Time 1. The Trust Scale is used to estimate secure attachment, as it assesses positive experiences of trust in the accessibility and responsiveness of the mother. It consists of 10 items (e.g., “I can count on my mother to help me when I have a problem.”) that are scored on a 4-point Likert scale ranging from 1 (*almost never or never true*) to 4 (*almost always or always true*). These 10 items were averaged to obtain a mean score of trust in maternal availability. In prior research, the Trust Scale score predicted children’s support seeking behavior during distress (Bosmans et al., 2015). In the current study, the internal consistency of trust in maternal availability (α = .80) was very good according to the criteria of DeVellis (2003).

#### Child Attachment Interview

The Child Attachment Interview (CAI; Shmueli-Goetz, Target, Fonagy, & Datta, 2008; Target et al., 2003) is a semi-structured interview that was adapted from the Adult Attachment Interview (AAI; George, Kaplan, & Main, 1985) for children between ages 8 and 15. Children are asked about recent attachment-related events and about the current relationships with their parents. In line with other studies using attachment narrative coherence as the main mode of measuring attachment (e.g., Borelli et al., 2016), we only used the coherence scale. This scale represents a dimensional measure of secure attachment (Venta et al., 2014). The CAI is a reliable and valid measure for attachment representations in middle childhood (Borelli et al., 2016; Shmueli-Goetz et al., 2008; Venta et al., 2014). Three certified CAI coders independently coded the same 30 CAI’s to establish rater agreement. After every 10 CAI’s, differences in ratings were discussed. The intraclass correlation coefficient (ICC) for coherence in the final 10 CAI’s was calculated in SPSS, using the two-way mixed model and absolute agreement for single measures. The ICC’s for the three coders ranged between .70 and .91, with an overall ICC of .81.

#### Middle Childhood Attachment Script Assessment

To measure secure base scriptedness in middle childhood, we used the Middle Childhood Attachment Script Assessment (Middle Childhood ASA; Waters et al., 2015) at Time 1. This is an adapted version of the Attachment Script Assessment for adults (ASA; Waters & Waters, 2006) for children between ages 9 and 12. Children were given a page with twelve prompt words organized into four columns that defined a beginning, middle and end for a potential story. Children had to tell stories in first person, pretending that the story was really happening. First, two stories about ordinary activities were told to practice (Snowy Day and Trip to the Park). After these two practice stories, three prompt word outlines for attachment-related stories (Scary Dog in the Yard, At the Beach, and Soccer Game) were presented. These three prompt word outlines are composed in a way that they elicit stories with a secure base script in children who possess this script. The presence of secure base script content in these transcribed attachment-related stories is scored on a scale from 1 (content inconsistent with secure base script) to 7 (rich secure base script content). Thus, a higher scriptedness score reflects a more elaborated secure base script content. Evaluation studies of the psychometric properties of the Middle Childhood ASA indicate that it is a valid and reliable measure of the SBS (Waters et al., 2015). The three coders were trained to code the MC ASA. Rater agreement was established by rating independently the same 20 MC ASA’s from this sample. Because rater agreement was not sufficient for the 20 dog stories, 20 additional dog stories were coded. ICC’s were calculated in SPSS using the two-way mixed model and absolute agreement for single measures. The ICC’s for the three coders ranged between .71 and .93 for the dog story, between .90 and .92 for the beach story, and between .82 and .90 for the soccer story. The final overall ICC’s for the three coders for were respectable to very good for the dog story (α = .79), the beach story (α = .91), and the soccer story (α = .85). After establishing sufficient interrater reliability, the three coders independently rated an equal number of stories. Finally, within each story, all stories were sorted per score and the score was adjusted if this was not equivalent in comparison with stories of the same score. The internal consistency of the three SBS stories (α = .70) was acceptable.

Using principal component analysis in SPSS, a common factor score was calculated based on the scores of the three measures for attachment (Coherence subscale of CAI, scriptedness score of MC ASA, and Trust subscale of PIML) (see Braet et al., 2013). This multi-method compound score reflects the common variance between the scores on the three measures of attachment, or in other words, the extent to which the three measures overlap on secure attachment. One factor with an eigenvalue higher than 1 (1.53) that explained 50.95% of variance was extracted with the Coherence subscale of the CAI loading .83 on this factor, the MC ASA .78, and the Trust subscale .47. As loadings above .30 suggest that items contribute relevantly to a latent factor (Field, 2005), all indicators were retained when calculating the factor score.

#### Distress

Children retrospectively reported at T2 and T3 on typically occurring stressors they experienced in the past three months of their lives in the Adolescent Life Events Questionnaire (ALEQ; Hankin & Abramson, 2002). In the current study, a shortened version of this questionnaire was administered with 40 items including 18 major life events and 22 daily hassles (Bastin, Mezulis, Ahles, Raes, & Bijttebier, 2015). For the analyses, we only used the daily hassles such as relationship difficulties (i.e., “You had an argument with a close friend”), and family problems (i.e., “You had a fight with parents over personal goals, desires, or choice of friends”). For this subset of items, children have to indicate on a 4-point Likert scale ranging from 0 (*never*) to 4 (*always*) how often a certain negative event has happened. These 22 items were averaged to obtain a mean score of distress. Supporting the validity of the ALEQ, ALEQ scores correlate highly to golden standard interview measures of stress (e.g., Bosmans et al., 2018).

#### Depressive symptoms

Children reported at T1, T2, and T3 on cognitive, affective and behavioral symptoms of depression that they had experienced in the past two weeks in the Children’s Depression Inventory (CDI; Kovacs, 2003; Dutch translation by Timbremont & Braet, 2002). For the 27 items of this questionnaire, participants have to indicate which from three descriptions fits best (e.g., “I feel like crying every day/many days/sometimes.”). Their answers are scored on a 3-point rating scale ranging from 0 to 2, with a higher score reflecting higher symptom severity. The scores on the 27 items were averaged to a mean score for self-reported depressive symptoms. Research has demonstrated that the CDI is reliable and valid (Kovacs, 2003; Saylor, Finch, Spirito, & Bennett, 1984) and can be used to discriminate children with major depressive disorders from non-depressed children (Kovacs, 2003). The internal consistency of self-reported depressive symptoms at T1 (α = .81), T2 (α = .82), and T3 (α = .84) was good.

### Procedure

To invite children and their mothers to our study, we distributed flyers with information about the study in the classrooms of the 4^th^, 5^th^ and 6^th^ grade. Invitation letters were distributed in schools nearby the two research locations where both mother and child were invited to come over for an assessment that was part of a broader study on attachment, cognitive vulnerabilities, and depression. A reward for participating (two movie theater tickets and the possibility of winning an mp3-player by participating in a lottery) was promised to each mother-child dyad for each year they participated. Each year, children and their mothers who wished to participate were contacted first by e-mail, and when there was no reaction, by phone. Upon arrival, active informed consent was obtained from both child and mother. Then, pictures of the mother for the ABT were taken with a digital camera. The measures for the child were administered in a fixed order in each wave among the other measures of the broader assessment. At T1: CAI, Trust scale, ABT, CDI, and MC ASA. At T2: CDI, ALEQ. At T3: CDI, ALEQ. 8.

A priori power analysis showed that testing a three-way interaction with 133 subjects yields a power of .95 to find a significant interaction with a small to medium effect size (*f^2^* = .10) and a power of .90 to find a significant interaction with the same effect size in 109 subjects. The (BLINDED) ethics committee approved the study design with protocol number S54974 (ML8907) and the title “The role and mechanisms of attachment-related attentional processing and the vulnerability for the development of depression”. The authors do not have competing interests.

### Plan of the Analyses

After conducting preliminary missing data and correlational analyses, we tested for age effects by bivariate correlations and for gender effects by independent samples *t*-tests. To test our three-way interaction hypothesis, we conducted hierarchical multiple regression moderation analyses in SPSS following the procedure of Aiken and West (1991), using the attachment factor score as predictor, and distress and ANE as moderators in the prediction of differences in depressive symptoms between T2 and T1 or T3 and T1. We controlled for depressive symptoms at T1. Sex and age at T1 were also entered as control variables to exclude possible influences on attachment and depressive symptoms (Agerup et al., 2015; Hankin et al., 1998; Twenge & Nolen-Hoeksema, 2002).^1^ To increase interpretability of the findings, all variables, except the dependent variable, were standardized first. The standardized regression weights and their effect sizes can be found in Tables 2 and 3 for relative changes in depressive symptoms from respectively T1 to T2 and T1 to T3. To generate data to plot the interaction effects and to examine the slopes of each association between attachment/distress/ANE and change in depressive symptoms at *M*+1*SD* and *M*-1*SD* of each moderator in the three-way interaction, the macro ‘PROCESS’ (Hayes, 2013) was used in SPSS. The values retrieved from the PROCESS macro are expressed in unstandardized regression weights.

## Results

### Preliminary analyses

All ABT trials in which the child identified the picture incorrectly (i.e., 7%) were excluded from analyses. At T1, there were 0.72% of the values missing on the questionnaire items, MC ASA stories, CAI coherence scale, and ABT. At T2 and T3, respectively 0.14% and 0.08% of the values were missing on the items of the CDI and the ALEQ. A mean score was not computed for scales with missing values. This resulted in 6% missing values on the variables and complete data for 109 participants. In the subsequent analyses, missing values were pairwise deleted.

In Table 1, descriptive statistics and zero-order correlations between all variables are presented. Depressive symptoms correlated positively over time. Distress from T2 and T3 correlated positively over time and with depressive symptoms. Trust correlated negatively with distress and depressive symptoms. The attachment factor score had a marginally significant negative correlation with depressive symptoms at T1. ANE had a marginally significant negative correlation with distress at T3. Furthermore, correlations between age at T1 and all the variables showed that age at T1 correlated positively with coherence, scriptedness, the attachment factor score, and with T3 depressive symptoms and distress at. Finally, we tested for sex differences on all the variables with independent samples t-tests. Girls scored higher on coherence, *t*(149) = 4.21, *p* < .001, scriptedness, *t*(148) = 2.73, *p* < .01, and the attachment factor score, *t*(145) = 4.15, *p* < .001.

**Table 1:** Means, Standard Deviations, and Correlation Coefficients Among the Variables (n = 121–157).

		1	2	3	4	5	6	7	8	9	10	11

1	Age T1	1										
2	ANE T1	.10	1									
3	Trust T1	.04	.02	1								
4	Coherence T1	.22**	.09	.18*	1							
5	Scriptedness T1	.22**	.11	.10	.45***	1						
6	Attachment FS T1	.24**	.10	.47***	.83***	.78***	1					
7	Distress T2	.06	–.08	–.27**	.09	.00	–.04	1				
8	Distress T3	.17†	–.16†	–.32***	.09	.10	–.00	.49***	1			
9	Depressive symptoms T1	.06	–.01	–.50***	–.05	.11	–.15†	.38***	.27**	1		
10	Depressive symptoms T2	.10	–.04	–.34***	–.00	–.01	–.13	.58***	.36***	.64***	1	
11	Depressive symptoms T3	.26**	–.06	–.24**	–.01	.09	–.04	.38***	.62***	.48***	.52***	1
	*M*	10.91	.04	3.58	5.73	3.90	0.00	0.67	0.78	0.24	0.26	0.27
	*SD*	0.87	.13	0.35	1.33	0.74	1.00	0.39	0.46	0.18	0.18	0.20

*Note*: ANE = attentional narrowing effect; Attachment FS = attachment factor score. † *p* < .10 * *p* < .05 ** *p* < .01 *** *p* < .001.

### Interactions between attentional narrowing around mother, attachment, and distress in the prediction of depressive symptoms

**Figure 3:**
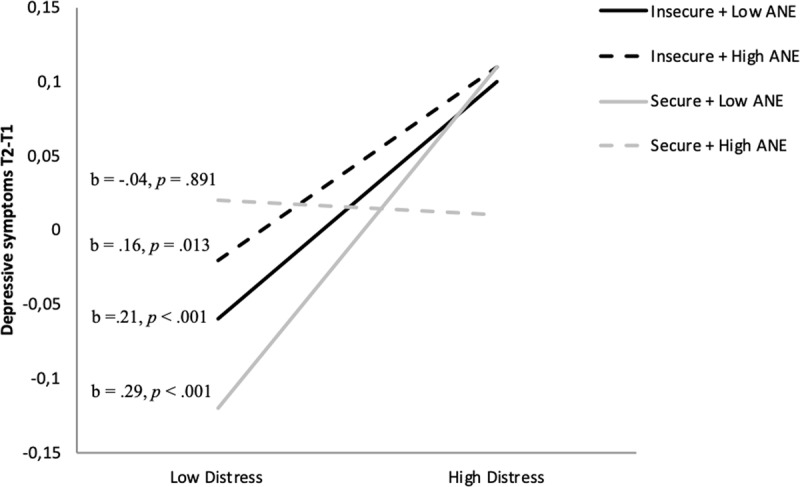
Prediction of change in depressive symptoms from T1 to T2, by the interaction between attachment (secure vs. insecure) and attentional narrowing around mother (high vs. low ANE) at T1 and distress (low vs. high) at T2, controlled for depressive symptoms at T1.

**The multiple regression analysis explaining depressive symptoms at T2** shows that the predicted three-way interaction effect between ANE at T1, the attachment factor score at T1, and distress at T2 was marginally significant (Table 2). This model explains 40% of the variance in change in depressive symptoms. Figure 3 shows, in line with our predictions, that at high levels of Distress (*M* + 1*SD*), the ANE × Attachment interaction marginally significantly predicted depressive symptoms at T2, *b* = –.19, *p* = .073. Only for children with higher levels of ANE and secure attachment at T1, the link between distress and changes in depressive symptoms at T2 was not significant. In all the other children, this link was significant. Moreover, further follow-up probing showed that for more distressed and more securely attached children, a more narrow attentional field around the mother was marginally significantly linked with a lower increase in depressive symptoms at T2, *b* = –.05, p = .062. These findings provided some first support for a protective effect of a narrow attentional field around mother for more securely attached children. No other effects were significant.

**Table 2:** Multiple Hierarchical Regression Analyses Predicting Change in Depressive Symptoms From Time 1 (T1) to Time 2 (T2) or Time 3 (T3) by ANE, Attachment and Distress (at T2 for T1 to T2 and at T3 for T1 to T3), and Their Two-, and Three-way Interactions.

	*T1 to T2*					*T1 to T3*				

	Δ*R*^*2*^	Δ*F*	*df*	*β*	*f*^*2*^	Δ*R*^*2*^	Δ*F*	*df*	*β*	*f*^*2*^

Step 1	.11	5.17**	3,120			.25	12.26***	3,109		
Age T1				.09	.03				.19**	.07
Sex T1				–.01	.00				.03	.00
Depressive symptoms T1				–.57***	.42				–.63***	.74
Step 2	.22	12.72***	3,117			.22	14.38***	3,106		
ANE T1				.07	.01				–.00	.00
Attachment T1				–.10	.01				–.07	.01
Distress				.42***	.24				.46***	.39
Step 3	.05	3.35*	3,114			.04	2.57†	3,103		
ANE T1 × Attachment T1				–.02	.00				.08	.01
ANE T1 × Distress				–.24**	.08				–.13	.03
Attachment T1 × Distress				–.06	.01				.06	.01
Step 4	.02	3.85†	1,113			.04	9.93**	1,102		.
ANE T1 × Attachment T1 × Distress				–.18†	.03				–.24**	.10

*Note*: ANE = attentional narrowing effect; Attachment = attachment factor score; Distress = at T2 for T1 to T2 and at T3 for T1 to T3. All reported *β* are values at Step 4 of analysis. † *p* = .053 * *p* < .05 ** *p* < .01 *** *p* < .001.

**Figure 4:**
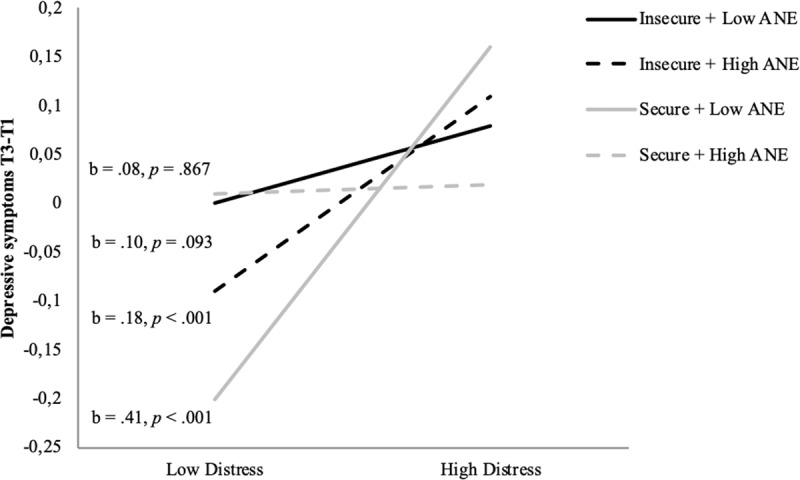
Prediction of change in depressive symptoms from T1 to T3, by the interaction between attachment (secure vs. insecure) and attentional narrowing around mother (high vs. low ANE) at T1 and distress (low vs. high) at T3, controlled for depressive symptoms at T1.

**The multiple regression analysis explaining depressive symptoms at T3** shows that the predicted three-way interaction effect between ANE at T1, the attachment factor score at T1, and distress at T3 was significant (Table 2).^2^ This effect survived Bonferroni correction as the p-value was smaller than .025. The model explains 40% of the variance in change in depressive symptoms. Figure 4 shows, in line with our predictions, that at high levels of Distress (*M* + 1*SD*), the ANE × Attachment interaction significantly predicted depressive symptoms at T3, *b* = –0.31, *p* < .05. Only for children with higher levels of ANE and secure attachment at T1, the effect of distress on depressive symptoms at T3 was not significant. More securely attached children (*M* + 1*SD*) showed significantly less change in T3 depressive symptoms at higher distress when they had a more narrow attentional field around their mother (reflected in higher ANE, *b* = –0.52, *p* < .05). Also in line with our predictions, at higher ANE and lower secure attachment, distress was significantly linked with increased depressive symptoms, whereas, at lower levels of ANE and lower secure attachment, the effect of distress did not reach full significance. However, in the less securely attached children (*M* – 1*SD*), differences in ANE were not significantly linked to change in depressive symptoms over time (*b* = 0.11, *p* = .566).

Interestingly, also at lower levels of distress (*M* – 1*SD*), the ANE × Attachment interaction significantly predicted depressive symptoms at T3, *b* = 0.54, *p* < .01. This effect reflects that more securely attached children with a lower ANE showed significantly more decrease in depressive symptoms than children with a higher ANE, *b* = 0.78, *p* < .001. For less securely attached children, change in depressive symptoms did not differ for different levels of ANE, *b* = –0.33, *p* = .159. The only remaining significant effect at low levels distress was a significant difference in changed depressive symptoms in more versus less securely attached children with low ANE, *b* = –0.10, *p* < .001).^3^

To assess the extent to which this three-way interaction effect was driven by the chronic or more recently increased levels of distress, we conducted two follow-up analyses with the PROCESS macro (due to which we can only report unstandardized *b*-values). In a first analysis, we focused on the impact of chronic distress. Therefore, we calculated a factor score across distress at T2 and T3. This factor score reflects the component of distress that was stable across both time points. One factor could be extracted with an eigenvalue >1 (1.488), explaining 74% of the variance, on which distress at T2 and T3 both loaded .86. When this factor score was included in the analysis, the three-way interaction became non-significant (*b* = –.06, *SE* = .14, *p* = .64). In a second analysis, we focused on the impact of recent distress. Therefore, we repeated the analysis with distress at T3 as moderator, but controlling for the effect of distress at T2. This way, we ensured that only the component of T3 distress that was not shared with T2 distress was taken into account when considering the three-way interaction. In this analysis, the three-way interaction remained significant (*b* = –.91, *SE* = .32, *p* = .006). This suggests that the three-way interaction specifically reflects effects of recently changed levels of distress.

## Discussion

The current study aimed to test the hypothesis that there is a three-way interaction between children’s attachment towards mother, exposure to distress, and the breadth of children’s attentional field around their mother to explain the development of depressive symptoms in adolescence. For this purpose, we carried out a longitudinal study, testing whether the three-way interaction predicts changes in depressive symptoms comparing baseline depressive symptoms to depressive symptoms in two subsequent waves one and two year later. Results showed that this interaction marginally significantly predicted relative changes in depressive symptoms at Time 2 and significantly predicted such relative changes at Time 3.

In both analyses, more securely attached children who had a narrower attentional field around their mother at baseline did not seem to develop depressive symptoms in response to later distress. If more securely attached children had a broader attentional field around their mother, distress was linked with increases in depressive symptoms over time. This supports the current study’s main hypothesis, suggesting that a narrower attentional field around the mother is a protective factor in securely attached children’s development. This finding is in line with prior research showing that more securely attached children with a narrower attentional field are least likely to display psychopathology (Bosmans et al., 2013; Claes et al., 2016; Van de Walle et al., 2017). The current study adds to these findings because now the role of distress in this association has been demonstrated more explicitly. Further adding to the literature is the longitudinal nature of the current study, due to which we now found support for the developmental value of securely attached children’s narrower attentional field around the mother. In light of the association we found earlier between a narrow attentional field around the mother and children’s support seeking behavior during distress (Bosmans et al., 2015), we assume that for more securely attached children a narrower attentional field facilitates support seeking during distress which in turn buffers against the maladaptive effects of distress.

Instead, at low levels of distress in adolescence, more securely attached children showed a significantly stronger decrease in depressive symptoms over time when they had a less narrow attentional field around their mother. This protective effect of a broad attentional field for less distressed securely attached children can be understood from the healthy effect of increased autonomy development at this age (Bosmans & Kerns, 2015). This is in line with other research showing that secure attachment gets increasingly linked to a wider attentional field around the mother as children grow older (Bosmans et al., 2017) and that securely attached children more easily attentionally disengage from mother in favor of attentional exploration (Dujardin et al., 2015). So, a broader attentional field around the mother might be highly adaptive in service of autonomy development, but only if children are not exposed to higher levels of distress later in their development.

In addition, and in line with our hypothesis, the analysis on Time 3 depressive symptoms indicated that children who were less securely attached were more at risk to develop depressive symptoms in response to adolescent distress when they had a narrower attentional field around mother. If they had a less narrow attentional field around mother, the link between distress and the development of depressive symptoms did no longer reach significance. The interpretation of this finding warrants caution. On the one hand, this finding supported our predictions and replicated prior cross-sectional research showing that a narrower attentional field around the mother is a risk factor when children are less securely attached (Bosmans et al., 2013; Claes et al., 2016; Van de Walle et al., 2017). On the other hand, this effect seemed less robust as it was not found in the analysis predicting Time 2 depressive symptoms. One reason might be that the strength of the three-way interaction effect increased as children developed due to which its effect on change in depressive symptoms became more observable. In a similar vein, Costello et al. (2003) suggested that the impact of risk factors on maladaptive development becomes increasingly visible as children transition into adolescence. So, it might be that a longer follow up would reveal even stronger negative effects of a narrow attentional field around the mother in less securely attached children.

Assuming that we found a valid effect, one can wonder why more insecurely attached children develop more depressive symptoms when they have a narrower attentional field around their mother. One explanation might be that this stronger focus on mother reflects insecurely attached children’s enhanced evaluation of whether or not they might seek maternal support during distress, which eventually delays or even disrupts proximity seeking. As a result, these children might depend on other, often less adaptive emotion regulation strategies to cope with distress. More specifically, due to lack of parental support, children might lack the strategies to adequately solve the problem that raises distress or to adequately regulate the negative emotions that are elicited by stressors (e.g., Folkman & Lazarus, 1988). As a result, these insecurely attached children might be more vulnerable to develop depressive symptoms.

Finally, at T3, we conducted follow-up analyses to assess the extent to which the three-way interaction was driven by more chronic distress or by more recent changes in distress levels. Results suggested that the three-way interaction was only significant when we included the variation in distress that was changed in comparison to distress as measured one year earlier. This finding is meaningful in light of our explanation that the three-way interaction reflects children’s (in)ability to find support that helps to regulate distress and buffers (or not) against its effects on the development of depressive symptoms. It seems to suggest that especially when distress recently increased, children’s resilience/vulnerability increases if they have a narrower attentional field around mother. This is presumably because increases in distress activate the attachment system and children’s desire to seek support (Bowlby, 1969), which might be facilitated in more securely attached children, and which might be suppressed in more insecurely attached children.

### Limitations

There were also limitations to the current study. Although the longitudinal design is a strength, no conclusions can be drawn about causality of the effects. Furthermore, all measures only used child report. This could have inflated the effects despite the multi-method design. However, a contra-indication for using other informants would be that parents are generally less accurate reporters of their child’s internal experiences (Angold et al., 1987; De Los Reyes & Kazdin, 2005). Moreover, the discrepancy between informants is related to individual differences in attachment (Borelli et al., 2017). Consequently, adding information from other informants to the analyses could have obscured the outcomes of the analyses.

Also, the current study focused on the child’s perception of the mother-child relationship only. Therefore, the findings may not be generalizable to father-child relationships. Although the mother-child relationship seems most relevant to the development of depressive symptoms (Allen, Hauser, Eickholt, Bell, & O’Connor, 1994; Brumariu & Kerns, 2010; Wenar & Kerig, 2005), some studies suggest that fathers and mothers influence children’s adjustment through different processes (Brumariu & Kerns, 2010; Papini & Roggman, 1992; Papini, Roggman, & Anderson, 1991). Hence, it may be relevant for future research to investigate whether attentional processing of father plays a similar role in children’s development.

Finally, the current use of a general population sample might limit the generalization of the results to clinical samples. In contrast, the mean of depressive symptoms and the percentage of children displaying (sub)clinical levels of depressive symptoms for the current sample were in line with prevalence rates that are on average found in general population samples of this age-group (Roelofs et al., 2010; Twenge & Nolen-Hoeksema, 2002). Future research could remedy these limitations by including other informants, and by targeting samples that are more heterogeneous or have other characteristics.

### Theoretical and Clinical Implications

In spite of these limitations, the current findings have important implications for our understanding of the role of information processing biases on the development of depressive symptoms. In general, attentional biases are often seen as always adaptive or maladaptive (e.g., Derryberry and Tucker, 1994). However, the current findings suggest – at least for attachment-related information processing biases – that the adaptive or maladaptive influence of such biases depends on factors such as differences in children’s attachment security and whether or not children are exposed to higher levels of distress. In addition, the current study contributes to our understanding why the link between attachment and the development of depressive symptoms is not stronger. We found not only that distress should be taken into account to find stronger links between less secure attachment and the development of depressive symptoms. We also found that more securely attached children can also be more vulnerable to develop depressive symptoms depending on their attentional focus on their mother.

Clinically, our results suggest that for insecurely attached children, it might be protective to repair the attachment relationship with their mother, but that the impact of children’s biases in the attentional processing of the mother should not be underestimated. Stimulating secure attachment development in adolescents has been successfully accomplished, for example, through Attachmnet-based family Therapy (e.g., Diamond et al., 2016). In addition, research also shows that it is possible to manipulate attachment-related information processing biases (Verhees et al., 2019) and that children can be trained to orient their attention to mother during distress which has a beneficial effect on their trust in maternal support (Bosmans et al., 2019). Combining such interventions could prove effective in the treatment of adolescent depressive symptoms (Verhees et al., 2019).

## Footnotes

Adding age and sex as control variables did not affect the results. For reasons of conciseness, we decided to only report the analyses that included these control variables.

For trust, the three-way interaction was significant in the prediction of depressive symptoms from T1 to T2 (*p* = .023) and from T1 to T3 (*p* < .001). For scriptedness, the three-way interactions were not significant in the prediction of depressive symptoms from T1 to T2 (*p* = .573) and from T1 to T3 (*p* = .948). For coherence, the three-way interactions were marginally significant in the prediction of depressive symptoms from T1 to T2 (*p* = .068) and from T1 to T3 (*p* = .071). Further, we repeated the analyses with a factor-score calculated using the T1 coherence and script scores. The hierarchical regression analyses revealed that the targeted three-way interaction did not reach significance when predicting change in depressive symptoms from T1 to T2, *β* = –.12, *p* = .122, nor when predicting change in depressive symptoms from T1 to T3, *β* = –.07, *p* = .297. This pattern of results supports Bosmans and Kerns’ (2015) assumption that the combination of the attachment measures at different levels of processing gives the best indication of the complex attachment construct.

We performed the hierarchical multiple regression analyses on the dataset with replaced missing values by the use of expectation maximization for T1, resulting in data for 133 participants. The expected three-way interaction effect between ANE at T1, the attachment factor score at T1, and distress at T3 significantly predicted depressive symptoms at T3 (*β* = –.21, *p* = .004). In the case of secure attachment, the effect of the ANE × Distress interaction significantly predicted depressive symptoms at T3 (B = –1.15, *p* = .002). In the case of insecure attachment, the effect of the ANE × Distress interaction did not predict depressive symptoms at T3 (B = 0.55, *p* = .117). The expected three-way interaction effect between ANE at T1, the attachment factor score at T1, and distress at T2 also significantly predicted depressive symptoms at T2 (*β* = –.18, *p* = .043), but the effect was less strong.
